# The promise of Immuno-oncology: implications for defining the value of cancer treatment

**DOI:** 10.1186/s40425-019-0594-0

**Published:** 2019-05-17

**Authors:** Howard L. Kaufman, Michael B. Atkins, Prasun Subedi, James Wu, James Chambers, T. Joseph Mattingly, Jonathan D. Campbell, Jeff Allen, Andrea E. Ferris, Richard L. Schilsky, Daniel Danielson, J. Leonard Lichtenfeld, Linda House, Wendy K. D. Selig

**Affiliations:** 1 Society for Immunotherapy of Cancer (SITC) Policy Committee, Replimune, Inc, 18 Commerce Way, Woburn, MA USA; 2 Georgetown University, 3970 Reservoir Road NW, Washington, D.C, USA; 3 Pfizer, Inc, 235 E 42nd St, New York, NY USA; 4 Amgen, Inc, One Amgen Center Drive, Thousand Oaks, CA USA; 5 Tufts University, 145 Harrison Ave, Boston, MA USA; 6 University of Maryland, 20 N. Pine St, Baltimore, MD USA; 7 University of Colorado, 12850 E. Montview Blvd, Aurora, CO USA; 8 Friends of Cancer Research, 1800 M St. NW, Washington, DC, USA; 9 LUNGevity Foundation, 228 S Wabash Ave, Chicago, IL USA; 10 ASCO, 2318 Mill Rd, Alexandria, VA USA; 11 Premera Blue Cross, 7001 220th St. SW, Mountlake Terrace, WA USA; 12 American Cancer Society, 250 Williams St, NW, Atlanta, GA USA; 13 Cancer Support Community, 734 15th St, NW, Washington, DC, USA; 14 WSCollaborative, 934 Bellview Rd, McLean, VA USA

**Keywords:** Immunotherapy, Immuno-oncology, Value, Patient experience, Patient reported outcomes (PROs)

## Abstract

The rapid development of immuno-oncology (I-O) therapies for multiple types of cancer has transformed the cancer treatment landscape and brightened the long-term outlook for many patients with advanced cancer. Responding to ongoing efforts to generate value assessments for novel therapies, multiple stakeholders have been considering the question of “What makes I-O transformative?” Evaluating the distinct features and attributes of these therapies, and better characterizing how patients experience them, will inform such assessments. This paper defines ways in which treatment with I-O is different from other therapies. It also proposes key aspects and attributes of I-O therapies that should be considered in any assessment of their value and seeks to address evidence gaps in existing value frameworks given the unique properties of patient outcomes with I-O therapy. The paper concludes with a “data needs catalogue” (DNC) predicated on the belief that multiple key, unique elements that are necessary to fully characterize the value of I-O therapies are not routinely or robustly measured in current clinical practice or reimbursement databases and are infrequently captured in existing research studies. A better characterization of the benefit of I-O treatment will allow a more thorough assessment of its benefits and provide a template for the design and prioritization of future clinical trials and a roadmap for healthcare insurers to optimize coverage for patients with cancers eligible for I-O therapy.

## Introduction: current clinical landscape

Compared with traditional cancer therapies, the approach described as Immuno-Oncology (I-O) therapy offers a more effective treatment alternative for some patients with cancer [1]. Rather than aiming treatments directly at the tumor, I-O therapies generally engage the immune system to recognize and eradicate tumor cells. Key features of immune-mediated therapy include specificity, breadth of response, and memory. These can contribute to complete tumor regressions, often providing more durable clinical outcomes and improved quality of life relative to cytotoxic chemotherapy, molecularly targeted therapeutics, and radiation, particularly in metastatic settings. The unique kinetics and properties of immunotherapy also result in different incidence and types of side effects, treatment length, and durability of response, as we describe in detail below. These differences need to be considered in studies of cost-effectiveness and value-based outcomes research, since I-O therapies are now approved by the U.S. Food and Drug Administration (FDA) in a variety of solid and hematologic malignancies, including melanoma, lung, kidney, bladder, head and neck, Merkel Cell, hepatocellular, certain gastrointestinal cancers, Hodgkin lymphoma, non-Hodgkin lymphoma, certain forms of leukemia, as well as in primary, site-agnostic tumors with Micro-Satellite-Instability High (MSI-Hi).

Most of the advances in I-O therapy to date have been demonstrated in patients with late-stage and metastatic cancer, but early results of adjuvant clinical trials using I-O therapies in patients with melanoma and lung cancer are promising. In addition, innovative approaches to patient selection, use of combinations, and sequencing of therapies lead to more patients benefitting from I-O therapy, expanding its potential impact. Typically, assessing impact of cancer therapeutics requires a minimum of five years follow-up to identify the benefit in overall survival. In melanoma, where I-O therapy has been available for the longest time, durable survival after I-O treatment has been confirmed [2].

There is urgent need to engage all stakeholders in maximizing I-O therapy’s impact for current patients and those diagnosed in the near term. Optimal I-O therapy utilization will require clinically appropriate quality benchmarks and an understanding of its true clinical and economic value.

### Immuno-oncology in the context of Cancer treatment

Until recently, the basic arsenal for treating cancer included surgery, radiation therapy, chemotherapy, and more recently, targeted therapy, sometimes in combination and often in sequence, to remove, reduce, eliminate or alleviate tumors. While these modalities often proved effective in producing durable remissions in patients with early, non-metastatic cancers, they generally failed to produce lasting benefit in patients with late-stage disease, except in certain leukemia, lymphomas, germ cell tumors and testicular carcinoma. Moreover, this multifaceted approach was often associated with serious negative consequences for patients, including disfigurement and a variety of treatment-related side effects caused by the total dose of radiation and the indiscriminate impact of cytotoxic agents on normal cells and physiologic functions.

Genomic studies conducted in the past two decades identified the molecular drivers of certain cancers and led to the advent of targeted therapies as an important additional pillar of the cancer therapy armamentarium. The current strategy generally follows a “one gene-one target” paradigm and is based on an assessment of specific gene mutations within an individual patient’s cancer. This approach, however, has been associated with high rates of acquired drug resistance largely through cancer cell upregulation of bypass pathways to circumvent the block in driver pathways. Many driver mutations have proven challenging for drug targeting [3].

Immunologists have evaluated a variety of approaches designed to stimulate and enhance the immune system’s response to tumors. The characterization of immune checkpoint pathways that can be targeted with immune-modulating antibodies energized a raft of drug development programs focused on inhibiting the effects of these immune checkpoints. The first of these immune checkpoint inhibitors, the anti-CTLA-4 antibody ipilimumab, was shown to produce durable survival in as many as 22% of patients with advanced melanoma, leading to FDA approval in 2011. Subsequent studies with a variety of PD1/PDL1 antibodies led to regulatory approvals as single agents and in combination with either anti-CTLA-4 or other agents in more than a dozen cancer indications [4].

The most recent frontier has leveraged chimeric antigen receptor (CAR) T cells as a successful treatment modality for patients with hematologic malignancies [5] and is a modality under clinical investigation for use in patients with some solid tumors. Oncolytic virus therapy, in which a virus can be used to infect and kill cancer cells, received approval in 2015 by the FDA of the treatment of patients with unresectable recurrent melanoma [6].

Emboldened by this progress, many pre-clinical and clinical activities are underway to advance and exploit therapeutic options through either exploiting means of activating antitumor immunity or crippling other mechanisms for evading immune destruction either alone or in combination with checkpoint inhibitors.

### What makes I-O therapy different? Scientific and clinical perspectives

#### Unique mechanisms of action

Cancer is basically a process of the patient’s own cells dividing rapidly and failing to die normally. For a cancer to become established in a host, the transformed cells must also develop mechanisms to avoid eradication by the immune system. Therapeutic manipulation can activate the innate immune system leading to cell death and, under appropriate conditions, innate and adaptive immunity leading to oncolysis while promoting long-term memory responses.

I-O therapy involves a fundamentally different approach from conventional chemotherapy, which unleashes an indiscriminate, static, and toxic direct attack on all cells – malignant and normal -- in hopes of damaging the cancer cells more than the host cells. Recent studies have suggested that cytotoxic chemotherapy and targeted therapy may also target stromal cells and immune cells within the tumor microenvironment [7, 8]. These observation suggest the potential for combining chemotherapy with IO agents with the goal not of killing as many tumor cells as possible, but rather to optimize immunologic clearance, which may allow for lower chemotherapy dosing. Because immunotherapy for cancer primarily relies on an indirect approach rather than a direct attack on cancer cells, the observed kinetics of response related to I-O therapies can be delayed [9] and, at times, the tumor may appear to be growing in the near term, when in fact the observed increase in volume is instead related to an inflammatory immune response that is working to eliminate the cancer [4].

#### Significant and increased durability of response

An adaptive immune response is characterized by the ability to persist, creating “immune memory” that, once effectively triggered by an immunotherapy, can enable the body to maintain an ongoing defense against a threat like a virus or a cancer cell expressing specific antigens, even after therapy is discontinued and perhaps for the lifetime of the patient. I-O therapies can also evolve over time, broadening and deepening anti-tumor immunity, preventing the cancer’s ability to escape through the selective growth of variants that can evade immune detection. The rapid co-evolution of tumor cells and immune responses may also result in immunoediting resulting in loss of antigen-specific immunity explaining, in part, IO drug resistance and the need to reconsider the pharmacologic drug class, dosing, schedule and combination to optimize anti-tumor activity [10].

Evidence of an effective and durable immune response against cancer dates back more than three decades, as high-dose interleukin 2 (IL-2) therapy produced durable responses with few relapses among approximately 10% of patients with advanced renal cell carcinoma (RCC) and melanoma [11]. These experiences demonstrated a unique hallmark of immunotherapy for the treatment of cancer: the flattening of the Kaplan Meier survival curve, in which a long, plateau of the curve represents durable responses that, for some patients, may extend throughout their lives.

With the advent of checkpoint inhibitors as single agents and in combination, dramatic results were first seen in patients with melanoma. The proportion of patients with metastatic melanoma experiencing objective responses increased to 20–22% with ipilimumab (anti-CTLA 4) treatment and 35–40% with anti-PD-1 agents, and above 50% with a combination approach [1].

Similarly, significant results with checkpoint inhibition approaches have yielded regulatory approvals of novel drugs and combination regimens, leading to new standards of care for patients with RCC, non-small cell lung cancer (NSCLC) [12], small cell lung cancer (SCLC) [13], bladder cancer [14], Merkel cell cancer [15], head and neck cancer [16], gastrointestinal cancer [17]and certain lymphomas [18]. Investigators are motivated by early success in identifying potential predictive biomarkers to select patients most likely to benefit (including programmed death ligand-1 or PDL1, and micro-satellite instability high or MSI-Hi), as checkpoint inhibition strategies are yielding even higher response rates in some tumors [19, 20]. Durable responses have also led to FDA approval of two CAR-T cell approaches for the treatment of acute lymphoblastic leukemia in children and young adults, and in certain forms of non-Hodgkin lymphoma in adults [21, 22].

#### Distinct side effect profiles

In general, immune checkpoint inhibitors have been associated with immune-related adverse events while CAR T cell treatment has been associated with cytokine release syndrome and neurologic toxicities. While serious adverse events are rare, mortality has been reported for patients receiving immune checkpoint inhibitors [23]. Nevertheless, I-O treatment has been suggested to have less impact on patients’ quality of life than conventional therapies [24], especially when adverse events are expeditiously managed early with corticosteroids and other immunosuppressive agents [25]. This parallels the experience with CAR-T research, in which cytokine release syndrome (CRS) was identified as an early potentially lethal clinical syndrome [26, 27], but an effective clinical management strategy was quickly identified [26], which did not appear to interfere with efficacy [28, 29], and actually led to a concomitant FDA-approved indication expansion for the anti-IL-6 monoclonal antibody, tocilizumab, since IL-6 is believed to be a major cytokine released in patients experiencing IO-induced cytokine release syndrome [30]. Research is ongoing to better define the most serious immune-related adverse events and identify patient characteristics most likely associated with them (recognizing that patient cohorts in most pre-approval studies did not fully reflect the general population) [31, 32].

### What makes I-O therapy different? Patient experience perspective

Many thousands of patients have been treated with immunotherapies in clinical trials and more recently, as standard of care. A holistic narrative is emerging about the patient experience with these novel therapies, providing important insights about how patients and caregivers perceive the value of these treatments. Patients often describe their experience with I-O agents in broader terms than the clinical outcome measures usually used in a trial. In addition to considering traditional effectiveness and safety measures like response rates, overall survival, and side effects, patients focus on the potential for limited treatment period duration, durability of response, the possibility of being “cured,” a more manageable side effect profile, and a better overall quality of life. Evaluating these aspects can provide important context and completeness for assessing the value of these therapies.

#### Limited treatment period duration: treatment free survival

Because I-O therapies act on the immune system, they may be effective if administered for a shorter period. As a result, many I-O treated patients experience significant “Treatment-free survival” (TFS), the period that occurs after treatment ends, and while the impact of the therapy endures, patients may not require other treatment(s) [33]. TFS provides an important opportunity for patients and their families to resume routine activities, travel, and generally approach their daily lives free from ongoing cancer treatment [34].

#### Effectiveness of therapy: return to productivity

There may also be financial benefits to individual patients, their families, and society that result from patients being able to return to work earlier and for longer periods of time while also reducing the need for additional or subsequent cancer treatments and perhaps less frequent medical tests and interventions. When effective, I-O treatment should boost productivity for many patients and may save individuals, families, and society considerable expenditures throughout the rest of their lives.

#### Impact of Treatment & Possibility of “cure”

While there is risk of serious toxicities associated with current I-O regimens, I-O therapies generally do not lead to the side effects commonly associated with cytotoxic chemotherapy such as nausea/vomiting, hair loss, and risks to fertility. In fact, the knowledge about 1) what side effects are likely to occur from I-O therapies and 2) that most can be managed in the near-term (by experiences providers) without impacting the effect of the cancer treatment, adds to patients’ current willingness to try them – especially when faced with few other potentially curative treatment options.

Late-stage patients facing the possibility of dying from their cancer often value the opportunity to pursue a hopeful gamble and receive a novel therapy that offers the potential for long-term disease control in a small percentage of patients rather than a treatment that offers potential benefit to a higher proportion of patients but for a shorter duration. Further, patients often will place a higher value overall on survival than their clinicians, who typically focus more on progression-free survival and managing patient’s treatment and disease related symptoms [35]. Of course, there are other factors at play in determining whether a patient has access to such hopeful gamble therapies, e.g. geographic access to healthcare provider expertise in IO delivery, drug availability, negative reimbursement incentives, high out-of-pocket expenses and others, raising important issues for society that are beyond the scope of this paper.

Reports of significant positive outcomes with I-O therapy for an increasing number of tumor types have fueled hope among patients for long-term survivorship and even cure in some cancers. This type of hope – especially for patients with dismal prognoses -- has been recognized to provide positive benefits to the patient’s quality of life [36] and is a powerful incentive for patients to seek access to these therapies, even while recognizing the longer odds of success. There is active debate within the oncology community about if and when to try immunotherapies when patients have few other valid options, even though the evidence is not yet conclusive about the potential benefit [37]. This may be especially important for patients with orphan cancers where clinical trials are lacking and where few approved agents are available.

### Assessing the value of I-O therapies

Economists frequently use the Incremental Cost Effectiveness Ratio (ICER) to assess and compare value in healthcare among available treatment options. ICERs are calculated by measuring or estimating the incremental costs and improvements in patient outcomes versus a therapeutic comparator through cost-effectiveness and cost utility models. The ICER measure is designed to be standardized across diseases. Health care payers often use the ICER to assess whether the improvements in patient health are worth the extra costs for one treatment versus another. For some, the ICER addresses an efficiency question, which can be helpful in a constrained resource environment. There are divergent views about the utility of the ICER measure in capturing value, especially given limitations in its ability to assess patient perspectives.

Currently, economic models are based on the metrics reported in the medical literature and are complicated by statistical uncertainty. These metrics generally describe treatment effects and adverse events reported in pivotal trials necessary to gain marketing approval by various national regulatory bodies, such as the FDA. While these metrics have rarely included patient-centered outcomes, the FDA has recently implemented a Patient-Focused Drug Development (PFDD) program to attempt to incorporate patient experience metrics into the regulatory pathway [38]. In the meantime, such outcomes are generally compiled during late stage development, especially for products that have gone through an accelerated approval.

The ability of current economic models to estimate ICERs is tied to the robustness of the data that are used to create the model itself. In oncology, economic modeling is challenging, in part because:

- Disease mechanisms vary by tumor type, genetic alteration, and location, that suggest heterogeneity of effect;
- Trial data are limited due to small study populations and relatively short follow-up; and
- Therapeutic effects of the therapy under investigation may be impacted by previous therapies a patient may have received.

These factors increase the uncertainty of economic model outputs and therefore negatively impact their capacity to precisely measure value in oncology. Various health technology assessment (HTA) bodies attempt to compensate for special cases such as disease severity, rare diseases, or end of life therapies, by adopting a lower ICER threshold by which ‘value’ is judged [39]. Others maintain the ICER threshold, evaluating all drugs against a common standard.

The definition of ‘value’ varies among stakeholders. For instance, patients and caregivers mostly overlap in how they define value, but subtle differences often exist between how patients differentially value returning to work or the impact of regaining their activities of daily living. Similarly, subtle but meaningful differences exist among how physicians, researchers, payers and employer groups define ‘value.’ In addition, the views of other stakeholders, such as drug developers, patients’ employers and family members are often not considered in the value assessment.

Within oncology, and specifically I-O, the assessment of value is made that much more difficult due to the principal impact of the therapy on landmark OS and the height of the plateau on the OS curve, rather than median PFS or OS, small numbers of patients assessed, and lack of long-term follow-up. These elements compound the uncertainty normally found within economic models [40].

Cost-effectiveness analysis (CEA) is an important tool when weighing the value of certain treatments using a common measure of health benefit. However, CEA is limited when accounting for other important aspects of ‘value’ to patients and may be misleading when long-term follow-up data on critical endpoints, such as overall survival, are not available. While these other aspects of value are arguably less important to decision makers allocating resources from a fixed budget, they should be accounted for when assessing value to patients and making decisions that may affect patient access.

#### Existing value frameworks and tools

Traditional clinical outcome measures, or clinical outcome assessments (COAs), in trials include overall survival (OS), progression-free survival (PFS) and objective response rate (ORR). These have long proved to be useful measures for assessment of cytotoxic chemotherapy, but a more complete assessment of the value of I-O requires identifying and measuring the impact of I-O therapy on patient’s lives. Some I-O therapy studies have shown significant improvement in overall survival without any impact on PFS, making the use of OS surrogates problematic in value frameworks that are not accounting for the potential differences in endpoint analyses.

A recent review by the ISPOR (International Society for Pharmacoeconomics and Outcomes Research) Special Task Force on US Value Frameworks has identified multiple value frameworks in the U.S. [41] In Europe, where HTA bodies are much more prevalent, there is less need for discrete value frameworks, but the European Society for Medical Oncology (ESMO) has created one based on “magnitude of clinical benefit.” [42] Others strive to be more patient-centered, emphasizing the patient experience [43]. In addition to understanding how each framework defines 'value', it is also important to consider that those designed by clinically-oriented bodies are meant to inform clinician-patient decisions, while those geared for payers are meant to inform payer and pharmacy benefit manager decision-making around coverage or formulary tiering.

The report of the Second Panel on Cost-Effectiveness in Health and Medicine (Second Panel) has defined four normative perspectives for consideration in evaluating value: 1) the payer perspective; 2) the health care sector perspective; 3) the health care sector with time cost perspective; and 4) societal perspective [44]. While each is scientifically valid and informative for their respective decision makers, the Second Panel recommended that analyses should include “reference cases” from the health care sector perspective and the societal perspective, which could be helpful in understanding how the value assessment informs a comparison within the therapeutic class or across therapeutic classes. Some stakeholders have noted a shortcoming in the Second Panel’s work, noting that it did not specifically call out patient perspectives in its report [45].

While some observers have criticized the recent value frameworks [46, 47], those meant to inform clinician-patient decisions do have elements of patient preference included in them, which may make the ‘value’ resulting from them reflective of an individualized assessment, and possibly then fit for informing individualized clinician-patient decisions. More payer-centric value frameworks also include elements of patient preferences but given the goal of informing population-level decision making, such value estimates are conducted at the average of a population. Thus, heterogeneity in individual patient preferences are often lost in these population-geared exercises.

### Identifying shortcomings of traditional metrics in assessing I-O value

#### Clinical efficacy measures for I-O

Because of the mechanistic differences between I-O therapies and traditional chemotherapy, conventional trial designs and endpoints generally do not fully capture the novel patterns of treatment response. This unique aspect of I-O suggests that longer-term assessment at multiple timepoints is needed to adequately evaluate outcomes [48]. Traditional parametric survival models used commonly to estimate long-term survival cannot adequately represent complex hazard functions and may not be appropriate for modelling the underlying mechanism of action associated with I-O treatments [49].

Recent work reported by the ISPOR Special Task Force in rare pediatric diseases presents some of the unique challenges in selection of clinical outcome assessments (COAs), and highlight the importance of developing uniform methods and metrics to capture relevant outcomes of interest for the I-O setting [50]. Additionally, recent work from the ISPOR Rare Disease Special Interest Group has identified several key challenges to research in rare diseases, which may be particularly relevant for I-O, and result in a lack of tailored health technology methods for rare disease treatments, as well as significant uncertainty for HTA authorities [51]. Many of the factors result from the evolving evidence base, including difficulties in establishing specific and sensitive diagnostic criteria, and evaluating the treatment effect (or heterogeneity of treatment effect). Combined with ethical challenges in designing appropriate clinical trials, insufficient knowledge of the natural history of the disease, and often poor patient recruitment for trials, the result is high levels of uncertainty in assessing value for these therapies. These uncertainties are factored into health technology assessments by global authorities, as comprising the level of certainty that is generally attributed to the value of a product. In addition, the model structure may not reflect the full patient experience, often failing to assess the value of treatment-free survival.

#### Safety assessments for I-O

While the long-term clinical and economic impact of safety monitoring with I-O therapy is not defined, current practice suggests that limited baseline screening and on-going laboratory monitoring with detailed clinical surveillance and patient education can identify adverse events early, allowing rapid intervention [52]. Whether this results in better compliance with planned treatment duration or prevents chronic toxicity is unknown. The optimal duration of treatment with I-O has also recently undergone considerable debate and discussion with some clinicians suggesting that early drug discontinuation may be possible without increasing rates of tumor progression [53].

An improved understanding of tumor immunology has led to new combination treatments, although it is unclear whether concurrent or sequential administration impacts outcomes. Further studies will focus on better defining effective combination regimens, treatment schedules and duration of therapy while refining safety monitoring measures that will allow appropriate patient management while limiting unnecessary diagnostic work-ups. These advances in limiting and mitigating toxicities should provide additional I-O relevant evidence to support better value assessments. There is a need for long-term follow up via accurate registries, capturing patient outcomes in community settings as well as academic medical centers.

#### PRO measures for I-O

One of limitations of reliance on the QALY within certain value frameworks is its primary dependence on survival endpoints (or improvements in OS and/or PFS) in determining the incremental cost per QALY gained for interventions that have OS and/or PFS primary endpoints in clinical trials [54]. Indeed, the ISPOR Special Task Force on Value Frameworks echoed the recommendation that cost-effectiveness analysis “as measured by cost per QALY [should serve] as a *starting point* to inform payer and policy maker deliberations” [55]. A natural question arises as to whether or not the QALY can be a comprehensive estimate of health outcome for the purposes of characterizing I-O therapies. Some cases of incremental cost-per-QALYs for I-O therapies suggest good value for money [56]. However, the question remains as to whether QALYs are sufficiently comprehensive to address the unique long-term outcomes for I-O, especially when compared to more traditional chemotherapy and targeted therapy regimens.

There is increasing interest in ‘going beyond QALYs’, to measure and systematically incorporate patient reported outcomes (PRO) in oncology [57–59], as there are signals (from markets outside the U.S.) that surrogate endpoints like PFS may not be closely associated with improvements in health-related quality of life in oncology clinical trials [60], or that current health-related quality of life instruments lack uniformity when applied across therapeutic areas [61]. While various work has suggested how to set standards for PRO use for cancer clinical trials with international standards [62], or in clinical trial protocols [63], there is more to be done before this work is ready for inclusion in value assessments. In fact, a recent FDA analysis has noted that health-related quality of life components most impacted by anti-PD-1/PD-L1 therapies (including disease symptoms, symptomatic toxicity and physical function) have been ‘variable,’ but that “these data, along with other important clinical data such as hospitalizations, ER visits and supportive care medications can help inform the benefit risk assessment for regulatory purposes.” [64]

In the U.S., the Centers for Medicare and Medicaid Services (CMS) has recently opened a National Coverage Determination (NCD) for Chimeric Antigen Receptor T-cell (CAR-T) Therapy for Cancers [65] and has focused on the PRO instruments themselves, and whether sufficient scientific evidence exists to support application of PROs to health outcomes research [66]. Presentations by the FDA and PRO experts provided optimism for several of the PRO instruments [67], and a final recommendation from the MEDCAC in the form of a proposed Decision Memo is expected in 2019 [68].

There is increasing interest in incorporating more patient centric elements in value assessments, especially as recent evidence appears to suggest an OS improvement among metastatic cancer patients who had PROs integrated into their routine care, compared to usual care [69]. While Basch had previously pointed out the lack of PRO data in existing value frameworks [70], he also argues for greater uniformity in how the PROs are incorporated into the value assessment for CAR-T cell therapies and to include patient representatives in consensus processes. While there seems to be increasing use of validated PRO instruments in oncology clinical trials, there are challenges to incorporating the PRO measures into existing value frameworks [71].

It is also challenging to weigh the different trade-offs between therapies in a class and the added layer of complexity associated with evaluating combination therapies. Likewise, there is the challenge of distinguishing between novel I-O therapies and their chemotherapy comparators, with the concept of treatment-free survival raising additional questions for researchers to address. An emphasis on integrating data collection regarding both PRO and quality of life (QOL) into modern I-O clinical trials will be important to developing benchmark metrics for understanding the impact of these measures related to specific drug agents and tumor types. The development of benchmark data will also provide a basis for comparisons to patient outcome data with more traditional cancer therapeutics.

### Recommendations for framework to develop value metrics for I-O: Data Needs Catalogue

This paper recommends the generation and synthesis [72] of evidence that will enable patients, health care providers, payers, and other stakeholders to make informed value-based decisions about I-O therapies (see Table 1). In addition to the clinical trials used for regulatory approval, more studies performed in real-world settings, e.g., pragmatic clinical trials, patient registries, health surveys, and administrative claims studies [73], would provide decision makers with a better understanding of the cost and benefits of treatments in the real world. As new data are generated, researchers must simultaneously work to incorporate them into value assessments.

**Table 1:** Assessment of conventional value metrics in evaluating I-O therapies

Conventional value metric (examples)	Why insufficent For I-O	Areas where new I-O value measures are needed (beyond QALY)
Clinical Efficacy Assessment *OS, PFS, ORR*	I-O therapies offer potential for durable response and due to delayed kinetics may not demonstrate early ORR or improvements in PFS	Milestone Survival; Treatment Free Survival
Safety Assessment	Late-stage cancer patients may be more willing to accept high risk of toxicity for possible benefit (durable response); long-term impact of adverse events not fully known	More nuanced evaluation of patient preferences based on their risk tolerance and profile; longer follow up studies post treatment
Patient Reported Outcome	Current measures fall short in measuring the value to patients of Treatment-free Survival; (extended time off treatment)	Treatment Free Survival impact on patient’s QoL; Hope for durable response
Economic Measures, e.g. Cost of ongoing treatment; Cost of treatment for side effects; cost of lost productivity	Typically focuses on patient-related expenses or drug cost during active treatment	Return to productivity; Economic benefit of Treatment-Free Survival, including reduced expenditures on ongoing treatment, scans and other follow up; Amortize costs over the longer horizon of benefit in a “cure-rate” model; consider other stakeholder fiscal impact

#### Develop better evidence, especially post-market

Post-market research is important to our understanding of the costs and real-world effectiveness of novel therapy approaches post launch. An important aspect of measuring real-world effectiveness is comparison of available treatment options in real world populations (i.e. comparative effectiveness). Thus, careful consideration for study design is needed not only to collect important elements of value but also to ensure that observed signals can be attributable to the I-O therapy.

#### Incorporate additional evidence into value assessments /modeling considerations

While evidence to support costs and real-world effectiveness estimates improves, researchers should advance models that support informed decisions. This may include, but is not limited to, increased modeling transparency [74], clearly outlining data and underlying assumptions used for calculations [75, 76], consensus on value elements [77] to incorporate into individual assessments, and continuous patient engagement [78, 79] throughout the process to ensure a patient-centric approach.

We recommend a concerted effort to develop models for looking beyond the median and conducting appropriate pre-planned sub-group analysis of the patients who see long-term benefit (e.g. “the Tail of the Curve” phenomenon, which within oncology, is seen by clinicians and patients as a defining hallmark of I-O). Table 2 describes considerations for such I-O specific elements to enhance a traditional ICER calculation.

**Table 2:** I-O specific elements to enhance traditional value calculations

Costs (numerator considerations)	Net Prices vs. List Prices	Wholesale acquisition costs may significantly overestimate the true cost of a drug. We recommend accounting for discounts and rebates where appropriate to reflect the true price paid for the new therapy.
	Consider alternate stakeholder perspectives	More research emphasis on a societal perspective – While many payers require a focus on the health sector specific costs, to fully understand the costs and benefits of a drug to society taking a societal perspective (accounting for caregiver costs, productivity gains/losses, etc.) in costeffectiveness analysis is warranted.
Effects (denominator considerations)	QALY	Many economic models are sensitive to the variations of the utility value used for each health state. We recommend engaging current or former patients as advisors to validate the assumptions made with the base case QALY inputs as well as the sensitivity analysis.
	Life Years	Conduct the same analysis with no QALY adjustment so that absolute mortality reductions can be easily reported for the decision-maker.
	Patient Specific	Identify other potential outcomes as denominators by engaging current and former patients. Addressing the outcomes that “matter” to patients can help decision-makers compare drugs within the same disease state for the specific population that it is impacting. Consider stratifying analyses based on risk tolerance of patient subpopulations.
Other factors (beyond the incremental cost effectiveness ratio)	Value of Hope	The ISPOR Special Task Force identifies this as an area needing more research to quantify, but it is conceptually intuitive and very relevant to IO. A cancer patient facing a terminal diagnosis may be willing to risk taking a more novel therapy if his or her chances include the possibility of durable response and even functional cure.
	Real Option Value	For a cancer patient, any innovation that can extend life (even at the same or worse quality of life) may give a patient a chance to live long enough for a new treatment to develop, possibly even a cure.
	Scientific Spillovers	New mechanisms of action may or may not benefit current patients, but we often fail to consider the steps in the path to future discovery. Without learning from the research in the 1950s, would we be here today with ~ 26 IO regimens benefitting thousands of patients?

### Future strategies for I-O analyses

While the field of I-O has advanced significantly in the past several decades, much more knowledge is needed to achieve a future where the potential benefit of these therapies can be maximized for the greatest number of patients. Key questions remain about how to select those patients who are most likely to respond to I-O therapy, how to combine I-O therapies with one another and with other treatment modalities, how to predict limit and mitigate I-O treatment related toxicities, how to reduce resistance to I-O therapies, how to use these therapies in newly defined standards of care and when to stop treatment.

Answers to these important questions – and addressing the important questions surrounding access to these therapies -- will help define and realize a promising vision for the future of cancer treatment, one that maximizes the potential of I-O therapy and further enhances its value to patients, their families, and society.

We envision a time when:

- Many more cancer patients will receive some form of I-O therapy during their treatment journey;
- We leverage patient reported outcomes, real world evidence and other tools to expand the knowledge base and continuously improve patient outcomes from I-O therapies;
- Careful patient selection ensures that treatments are provided only to those patients most likely to benefit;
- The numbers and cancer profiles of patients who are likely to benefit has expanded;
- Potential resistance to I-O therapy is reduced and we succeed in turning previously non-immunogenic cancers into ones that can respond to I-O therapy;
- The benefits are established for I-O therapy in the adjuvant and neo-adjuvant settings, thereby reducing the incidence of late-stage cancers; and
- Cancer can become a treatable and even curable set of diseases [80] with combination approaches that include I-O leading to maximized therapeutic equations for every cancer and a resulting favorable economic impact for patients, their families and society.
